# Implementation and Impact of Postdischarge Support of Cardiovascular Patients Using Text Messages The HeartHealth Program

**DOI:** 10.1016/j.jacadv.2025.102017

**Published:** 2025-08-06

**Authors:** Brodie Sheahen, Simone Marschner, Haeri Min, Aravinda Thiagalingam, David Pryce, Robert Denniss, Pramesh Kovoor, Dylan Wynne, Anupama Balasuriya Indrawansa, Clara K. Chow

**Affiliations:** a Westmead Applied Research Centre, Faculty of Medicine and Health, The University of Sydney, Westmead, New South Wales, Australia; b Department of Cardiology, Westmead Hospital, Westmead, New South Wales, Australia; c Westmead Hospital, Westmead, New South Wales, Australia

**Keywords:** cardiovascular disease, digital health, health care utilization, patient support, SMS, text messaging

## Abstract

**Background:**

The HeartHealth program is a text message support program for cardiovascular patients in Western Sydney, Australia. It comprised regular semipersonalized text messages providing cardiovascular disease information, advice, and support over a 6-month period.

**Objectives:**

This study aimed to examine the impact of the HeartHealth program on health care service utilization.

**Methods:**

This is an observational study that compared hospitalization rates in patients who enrolled in the HeartHealth program to patients who did not. Patients ≥18 years old who attended cardiology clinics or were discharged from the cardiology unit at Westmead Hospital between April 2020 and April 2022 were invited to participate in HeartHealth. The primary outcome was the incidence rate of all-cause health care utilization events in the 6 months postdischarge. All analyses were adjusted for demographic and comorbidity variables.

**Results:**

11,542 patients were included in the analysis (intervention: 4,324, control: 7,218), with a mean age of 58.8 ± 17.0 years, and 60.7% (7,003/11,535) were male. HeartHealth participants had lower rates of health care service events compared to control participants (number of events per patient: 0.43 intervention vs 0.52 control; adjusted rate ratio [RR]: 0.91 [95% CI: 0.83-0.99]; *P* = 0.033). There was a greater reduction in health care utilization events in older (>65 years old) compared to younger patients (RR: 0.82; 95% CI: 0.72-0.93 vs RR: 0.98; 95% CI: 0.87-1.10, respectively; interaction *P* = 0.043). HeartHealth participants reported that the program improved diet, physical activity levels, and medication compliance.

**Conclusions:**

This analysis demonstrates the potential of text message-based postdischarge support for cardiovascular disease patients to decrease their need for health care service utilization for all medical causes.

Cardiovascular disease (CVD) is the leading cause of death and disease burden globally.^1^ In Australia, approximately 1.2 million adults live with CVD, and CVD is responsible for about 25% of all deaths.^2^ Targeting modifiable risk factors is key to primary and secondary prevention of CVD.^3^^,^^4^ But many barriers to good risk factor control exist. These include poor health literacy,^5^ poor access to treatments and services due to geography, time-pressures, and socioeconomic and cultural barriers.^6^, ^7^, ^8^, ^9^ Personalized and customized support can address these barriers but is often not cost-effective. Digital health utilizes information and communication technologies in medicine and other health professions to manage illness and health risks, which has gathered momentum due to the potential of improving health care access, decreasing inefficiencies, providing more personalized health care, and lowering the costs.^10^

In the area of cardiology, there are now many examples of digital health interventions that have shown benefits in managing a range of cardiac conditions and CVD prevention.^11^ There is evidence that text message-based CVD secondary prevention programs have improved CVD risk factors^12^, ^13^, ^14^ and are cost-effective.^15^ Similarly, text-messaged interventions improve CVD risk factors in primary prevention.^16^ For example, patients receiving a texting program across various studies were twice as likely to quit smoking,^17^ physical activity levels increased,^18^ blood pressure reduced,^14^ and weight management improved.^19^

While the data to date demonstrates clear improvements in important chronic disease risk factors, there is little information on the impact of a postdischarge text messaging support program on health care utilization within cardiovascular patients. In April 2020, we introduced HeartHealth in Western Sydney, offering it to all patients who were discharged from cardiovascular services or clinics. The current study aims to describe the implementation of this program and the impact of the program on health care utilization.

## Methods

### HeartHealth program

The HeartHealth program provides postdischarge support for cardiovascular patients and encourages cardiovascular healthy lifestyle change through prompts, education, and supportive tips tailored to participants’ characteristics. Participants are offered via mobile phone text message to enroll in the program following discharge from an inpatient cardiology setting or following attendance at an outpatient cardiology clinic. Program content is delivered via mobile phone text message, and additional content is embedded in text messages with weblinks and videos. About 4 messages are sent per week over a 6-month period. Program content is tailored using responses to a survey sent at program enrollment (eg, hypertension, diabetes mellitus, hypercholesterolemia, smoking status, and vegetarian status). Overall program content was curated by Westmead Cardiology Department clinicians, patients, and researchers, with the core structure based on the previously published TEXT ME program.^12^ Message content development was based on a range of theoretical frameworks as previously described.^20^ The program content is actively reviewed and updated. Key topics addressed include diet, physical activity, general cardiovascular health, medication compliance, and key risk factors as applicable to the participant; examples of SMS messages delivered are outlined in Box 1. Additional content, such as specific messages related to COVID-19, was added across the implementation period. The electronic management and application of the varying clinical algorithms enabling customization of content is through a secure cloud-based digital platform, “TextCARE” developed by the team at Westmead Applied Research Centre, University of Sydney.

**Box 1: Examples of Program Messages**

**Table:** 

Diet
Hi [NAME], did you know the goal for an average adult is to consume <1 teaspoon of salt per day? To learn more visit [URL]
Hi [NAME] try leaving a plate of chopped fruit on the table to graze on after dinner.
Physical activity
Have you exercised this week [NAME]? We recommend at least 30 mins of moderate-intensity activity a day. If 30 mins is too much at once, do 10 min at a time until you reach 30.
Hi [NAME], activity can be accumulated in shorter bouts of 10 mins each. Look at these tips: [URL link]
General cardiovascular information
It is hard to remember to take tablets every day! Try & make it easier—put them next to your toothbrush or somewhere easy to remember.
Do you know the warning signs of heart attack [NAME]? To learn more visit [URL]
Optional - Smoking
[NAME], when quitting smoking - enlist your doctor's help, try a nicotine chewing gum, patches, or inhaler.
Hi [NAME] smoking increases the risk of heart disease by up to 6 times. To learn more visit [URL]
Other messages - eg, COVID-19
More people are working from home or isolating themselves, and social isolation is associated with heart-unhealthy behaviors. It is good to be aware of this; try to talk with others every day and try to keep active.
COVID-19 vaccination reduces the risk of being hospitalized. Read here for useful info on how being vaccinated protects you. [URL]

### Study design

This is an observational study design comparing patients who received the HeartHealth program to patients who did not receive the program and examining aspects of program implementation. The evaluation used local health district electronic medical records (EMRs) data and postprogram survey data. The study design arose as a pragmatic evaluation of a program supported by local health services for implementation during the COVID-19 pandemic. Patients were invited to the HeartHealth program either following attendance at a Westmead Hospital outpatient rapid access cardiology clinic or following discharge from an inpatient cardiology admission at Westmead Hospital. The study comprises a case-controlled comparison of participants who enrolled in HeartHealth (intervention cohort) with participants who did not enroll (control cohort). This in-time comparator group was selected to avoid a pre-post comparison with the period prior to the COVID-19 pandemic, as studies have shown the impact of COVID-19 on hospitalization and emergency department presentation rates.^21^^,^^22^

### Setting

Western Sydney Local Health District (WSLHD) is comprised of 5 public hospitals that serve approximately 1.1 million residents.^23^ It includes a diverse population where 46.8% of residents are born overseas and 50.3% speak a language other than English.^24^ Ethics approval for this study was granted by the WSLHD Human Research Ethics Committee.

### Enrollment and eligibility

The HeartHealth program was initiated in April 2020 and continues to be deployed. The HeartHealth program was initially deployed through cardiovascular services at Westmead Hospital, the largest cardiology department in the district, which also provides most of the public cardiology outpatient services. Patients post-hospital discharge from a cardiology admission or who had recently attended outpatient cardiology clinics are offered enrollment in HeartHealth with a text message asking them to opt-in and consent to the program. For patients who do not respond to this initial text message, an attempt to reach the patient is made by phone call by a staff member working part-time on the program.

### Data collection

Patient-level variables included age, gender, country of birth, preferred language, and comorbidities. Emergency department (ED) variables included presentation reason, presentation type (planned/unplanned), date/time of presentation, and date/time of discharge. Hospital inpatient variables, including admission reason, admission type (planned/unplanned), date/time of admission, and date/time of discharge were obtained from electronic medical records. The analysis dataset linked records to public ED data, inpatient encounters, and patient-level variables across WSLHD services between April 21, 2020, and April 1, 2022. EMR data were linked, processed, and provided by the Business Analytics Service at Westmead Hospital to the Westmead Applied Research Centre, University of Sydney research team at Westmead for analysis.

All participants were asked to complete an assessment survey at the end of the intervention. This survey had 4 separate quantitative components. The survey used a 5-point Likert scale assessing program acceptability, usefulness, program engagement, and changes in lifestyle behaviors. The survey was distributed via REDCap.

HeartHealth program data of patient reasons for nonenrollment were collected. Patients were initially invited to the HeartHealth program via a text message. If patients chose not to opt-in to the HeartHealth program, they were not asked to provide a reason as to why. However, a recent adaptation made to program enrollment process was that those patients who did not respond to the initial program invite (did not opt in nor opt-out) received a phone call from the HeartHealth team to reinvite the patient to the program. Here, if patients did not opt into the program, they were asked to provide a reason.

### Definitions and outcomes

A hospital admission event was defined as the participant requiring admission to the hospital as an inpatient; however, it did not include admission to the ED. An ED event was defined as the participant presenting to the ED without subsequent admission to the hospital as an inpatient.

Reasons for ED presentation and hospital admission were classified as either an all-cause, all CVD-cause, or unplanned CVD-cause. An event was classified as unplanned if the EMR presentation or admission type was categorized as “Emergency,” “Unplanned Return Visit for continuing condition,” “Disaster,” or “Dead on arrival.” Conversely, an event was classified as planned if the presentation or admission status was categorized as either “Non-Emergency/Planned,” “Return visit - planned,” “Prearranged Admission,” “Maternity/Newborn,” or “Regular Same Day Planned Admissions.” A composite of health care utilization events is where ED presentation events and hospital admission events were grouped together. Events were captured between 0 and 6 months from intervention commencement. The subsequent event classifications are defined in Table 1.

**Table 1:** Classification of Health Care Utilization Events

Event Classification	Definition
All-cause health care utilization events	Composite of ED presentations and/or hospital admissions for any cause
All-cause ED presentations	All ED presentations for any cause
All-cause hospital admissions	All hospital admissions for any cause
All CVD health care utilization events	Composite of all (planned or unplanned) ED presentations and/or hospital admissions for a CVD cause
All CVD ED presentations	All (planned or unplanned) ED presentations for a CVD cause
All CVD hospital admissions	All (planned or unplanned) hospital admissions for a CVD cause
Unplanned CVD health care utilization events	Unplanned ED presentations and/or hospital admissions for a CVD cause.
Unplanned CVD ED presentations	An unplanned ED presentation for a CVD cause
Unplanned CVD hospital admissions	An unplanned hospital admission for a CVD cause

CVD = cardiovascular disease; ED = emergency department.

### Study outcomes

The primary outcome was the number per patient of all-cause health care utilization events (composite of ED presentations and/or hospital admissions for any medical cause) within 6 months of program commencement. We selected 6 months to match the duration of the program. The secondary outcomes were the number per patient of each of the following: unplanned CVD hospital admissions, ED presentations, and health care utilization events; incidence rates of all CVD hospital admissions, ED presentations, and health care utilization events; and incidence rates of all-cause hospital admissions and ED presentation. The main reasons for hospitalization have been categorized according to the hospital coding systems of this outcome. Coding of these outcomes is part of usual processes in the health system. They would not have been done with knowledge of patients having received or not received the HeartHealth program. For the purposes of this study, we took hospital-coded definitions, and the outcomes were not independently adjudicated.

Furthermore, secondary outcomes, including change in healthy diet, physical activity, motivation to lead a healthy lifestyle, and medication adherence, were defined as agreeing or strongly agreeing to statements on each item.

### Statistical analysis

Continuous variables are presented as mean ± SD based on Shapiro-Wilk test of normality. Categorical variables are presented as frequencies and percentages. Univariable and multivariable analyses were performed to explore factors associated with the study outcomes. The outcomes of incidence rates for health care utilization events, ED presentations, and hospital admissions for all-cause events, all CVD events, and unplanned CVD events were assessed using an adjusted Poisson regression analysis. In addition, an interaction term was added to the model to assess differing treatment effects across age, gender, place of recruitment, and English as the preferred language. Any imbalances in demographic and comorbidity variables will be adjusted for in the analyses. All adult patients (≥18 years) offered the HeartHealth program between April 21, 2020, and April 1, 2022, were included in the study. Patients in the intervention group were excluded from analysis if they did not complete the entire 6-month program duration, and patients were excluded if they had reoccurring hospital admissions for dialysis treatment. Sensitivity analyses were done using the same models, however excluding the ‘Born in Australia/New Zealand’ and ‘English as preferred language’ variables, as these were the only variables with >1% missing data. All statistical tests were 2-tailed, and a 5% significance threshold was maintained. Statistical analysis was undertaken using R statistical software (V4.2.0).

## Results

**Figure 1:**
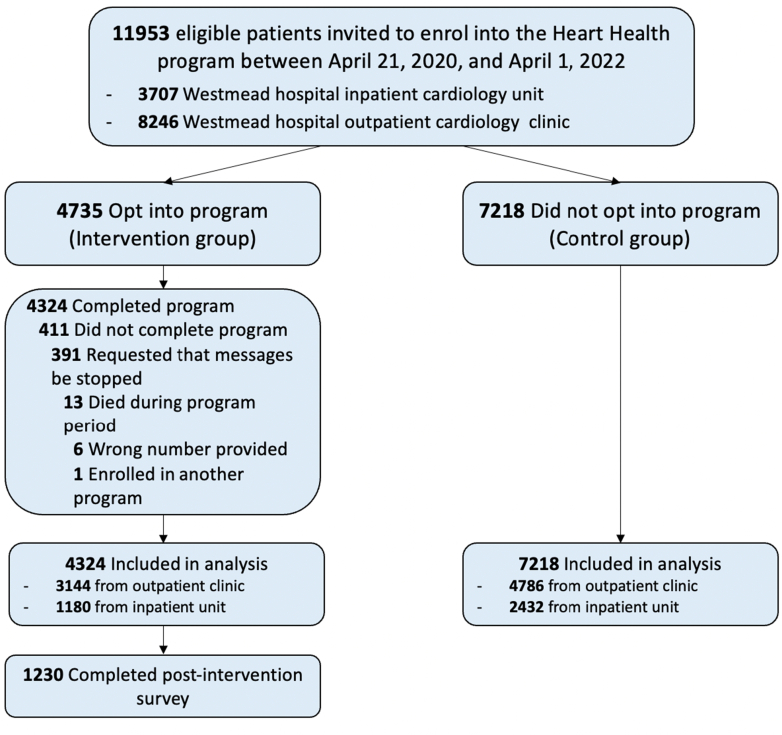
Enrollment of Participants in the HeartHealth Program Enrollment of participants in the HeartHealth program, including those invited to the program, those who did not enroll, and those who did not enroll and completed the end-of-program survey.

Between April 21, 2020, and April 12, 2022, 11,953 patients were invited to enroll into the HeartHealth program, 39.6% (4,735/11,953) of patients opted into the program and 36.2% (4,324/11,953) of patients completed the full 6-month program, which is an 8.7% dropout rate. Data waere linked for 11,542 patients (intervention group: 4,324, control group: 7,218). Overall, the mean age was 58.8 (SD: 17.0) years (Figure 1), 60.7% (7,003/11,535) were male, 68.7% (7,930/11,542) were from an outpatient cardiology clinic, English was the preferred language in 78.3% (8,377/10,701) of participants, and 45.5% (4,854/10,661) were born in Australia or New Zealand. Data completeness was high for all variables, with <1% missing for all variables except for patient preferred language (841/11,542; 7.3%) and country of birth (881/11,542; 7.6%). There was no missing data for outcome variables or comorbidity variables, as these were derived from routinely collected hospital data (Table 2).

**Table 2:** Patient Demographic and Comorbidity Details

	Control (n = 7,218)	Intervention (n = 4,324)	Overall (N = 11,542)
Age, y	59.9 (17.5)	56.7 (15.8)	58.8 (17.0)
Sex			
Male	4,451/7,215 (61.7%)	2,552/4,320 (59.1%)	7,003/11,535 (60.7%)
Female	2,764/7,215 (38.3%)	1,768/4,320 (40.9%)	4,532/11,535 (39.3%)
Missing	3	4	7
Place of recruitment			
Clinic	4,786/7,218 (66.3%)	3,144/4,324 (72.7%)	7,930/11,542 (68.7%)
Hospital	2,432/7,218 (33.7%)	1,180/4,324 (27.3%)	3,612/11,542 (31.3%)
Country of birth			
Australia and New Zealand	3,002/6,697 (44.8%)	1,852/3,964 (46.7%)	4,854/10,661 (45.5%)
Americas	88/6,697 (1.3%)	51/3,964 (1.3%)	139/10,661 (1.3%)
North Africa and the Middle East	894/6,697 (13.3%)	364/3,964 (9.2%)	1,258/10,661 (11.8%)
Northeast Asia	398/6,697 (5.9%)	250/3,964 (6.3%)	648/10,661 (6.1%)
Northwest Europe	357/6,697 (5.3%)	184/3,964 (4.6%)	541/10,661 (5.1%)
Oceania (not including Aus/NZ) and Antarctica	255/6,697 (3.8%)	152/3,964 (3.8%)	407/10,661 (3.8%)
Southeast Asia	358/6,697 (5.3%)	243/3,964 (6.1%)	601/10,661 (5.6%)
Southern and Central Asia	688/6,697 (10.3%)	598/3,964 (15.1%)	1,286/10,661 (12.1%)
Southern and Eastern Europe	564/6,697 (1.4%)	208/3,964 (5.2%)	772/10,661 (7.2%)
Sub-Saharan Africa	93/6,697 (1.4%)	62/3,964 (1.6%)	155/10,661 (1.4%)
Missing	521	360	881
English as preferred language	5,109/6,722 (76%)	3,268/3,979 (82.1%)	8,377/10,701 (78.3%)
Missing	496	345	841
Hypertension	1,001/7,218 (13.7%)	422/4,324 (9.8%)	1,423/11,542 (12.3%)
Hypercholesterolemia	81/7,218 (1.1%)	34/4,324 (0.8%)	115/11,542 (1%)
Diabetes mellitus	1,100/7,218 (15.4%)	471/4,324 (10.9%)	1,581/11,542 (13.7%)
Obesity	582/7,218 (8.1%)	256/4,324 (5.9%)	838/11,542 (7.3%)
Alcohol misuse disorder	63/7,218 (0.9%)	23/4,324 (0.5%)	86/11,542 (0.74%)
Smoker	1,429/7,218 (19.8%)	599/4,324 (13.8%)	2,028/11,542 (17.6%)
Heart failure	2,362/7,218 (32.7%)	1,099/4,324 (25.4%)	3,461/11,542 (30%)
Ischemic heart disease	1,441/7,218 (20%)	656/4,324 (15.2%)	2,097/11,542 (18.2%)
Acute coronary disease	1,045/7,218 (14.5%)	470/4,324 (10.9%)	1,515/11,542 (13.13%)
Atrioventricular block	285/7,218 (3.9%)	109/4,324 (2.5%)	394/11,542 (3.4%)
Atrial fibrillation	1,176/7,218 (16.3%)	538/4,324 (12.4%)	1,714/11,542 (14.8%)
Supraventricular tachycardia	223/7,218 (3.1%)	175/4,324 (4.1%)	398/11,542 (3.4%)
Ventricular tachycardia and fibrillation	339/7,218 (4.7%)	178/4,324 (4.1%)	517/11,542 (4.5%)
Sick sinus syndrome	149/7,218 (2.1%)	67/4,324 (1.6%)	216/11,542 (1.9%)
Cardiac arrest	82/7,218 (1.1%)	37/4,324 (0.9%)	119/11,542 (1%)
Pulmonary embolism	146/7,218 (2%)	64/4,324 (1.5%)	210/11,542 (1.8%)
Cerebral infarction	210/7,218 (2.9%)	100/4,324 (2.3%)	310/11,542 (2.7%)
Transient ischemic attack	149/7,218 (2.1%)	66/4,324 (1.5%)	215/11,542 (1.9%)
Peripheral vascular disease	67/7,218 (0.9%)	18/4,324 (0.4%)	85/11,542 (0.7%)
Chronic obstructive pulmonary disease	445/7,218 (6.2%)	141/4,324 (3.3%)	586/11,542 (5.1%)
Chronic kidney disease	552/7,218 (7.6%)	199/4,324 (4.6%)	751/11,542 (6.5%)
Asthma	352/7,218 (4.9%)	185/4,324 (4.3%)	537/11,542 (4.6%)
Thyrotoxicosis	54/7,218 (0.7%)	26/4,324 (0.6%)	80/11,542 (0.7%)
Obstructive sleep apnea	112/7,218 (1.6%)	40/4,324 (0.9%)	152/11,542 (1.3%)

Values are mean (SD) or n/N (%).

Uptake of the intervention was slightly greater in females (intervention 1,768/4,320; 40.9% vs control 2,764/7,215; 38.3%), in younger people (mean age intervention 56.7 years and control 59.9 years) and among people with English as a preferred language (intervention 3,268/3,979; 82.1% vs control 5,109/6,722; 76.0%). The control group cohort generally had a worse risk factor profile than the intervention group (Table 2). There were statistically significant discrepancies between the cohorts in regard to the following demographic and comorbidity variables: age, gender, “Born in Australia/New Zealand,” “English as the preferred language,” site of recruitment (post-hospital discharge or from outpatient clinic), history of CVD and particular comorbidities (hypertension, hypercholesterolemia, diabetes mellitus, obesity, alcohol misuse disorder, smoker, heart failure, ischemic heart disease, acute coronary disease, atrioventricular block, atrial fibrillation, supraventricular tachycardia, ventricular tachycardia and fibrillation, sick sinus syndrome, cardiac arrest, pulmonary embolism, cerebral infarction, transient ischemic attack, peripheral vascular disease, chronic obstructive pulmonary disease, chronic kidney disease, asthma, thyrotoxicosis and obstructive sleep apnea. All of these covariates could potentially impact the outcome variables; therefore, all analyses were adjusted for these covariates.

### Incidence of health care utilization events

There was a lower rate of the primary outcome of all-cause health care utilization events in the intervention group compared to the control group, and this remained significant after adjusting for covariates (rate ratio [RR]: 0.91 [95% CI: 0.83-0.99]; *P* = 0.033). Incidence rates for the outcomes of CVD events and unplanned CVD events were numerically lower in the intervention group but did not remain statistically lower after adjusting for covariates. (Table 3). All-cause hospitalization rates were lower in the intervention group (RR: 0.91 [95% CI: 0.84-1.00]; *P* = 0.044), compared to the control group. There were no statistically significant differences in unplanned CVD hospitalization or any CVD hospitalization (Table 3). There were no differences in ED presentations between cohorts for unplanned CVD cause events (RR: 1.09 [95% CI: 0.90-1.33]; *P* = 0.360); all CVD-cause events (RR: 1.10 [95% CI: 0.91-1.33]; *P* = 0.339) and all-cause events (RR: 0.99 [95% CI: 0.85-1.14]; *P* = 0.816) during 0 to 6 months from intervention commencement (Table 3).

**Table 3:** Adjusted Incidence and Adjusted Rate Ratio of Health Care Utilization Events (ED Presentation and/or Hospital Admissions), Hospital Admissions, and ED Presentations Over a 6-Month Period

	Treatment Group			
	Control (n = 7,218)^a^	Intervention (n = 4,324)^a^	Rate Ratio (95% CI)	*P* Value
Health care utilization events				
All-cause health care utilization events	3,756 (0.52 [0.49-0.55])	1,856 (0.43 [0.40-0.46])	0.91 (0.83-0.99)	0.033
All CVD health care utilization events	1,637 (0.23 [0.21-0.24])	946 (0.22 [0.20-0.24])	1.06 (0.95-1.17)	0.307
Unplanned CVD health care utilization events	1,173 (0.16 [0.15-0.18])	626 (0.14 [±0.13-0.16])	1.02 (0.89-1.16)	0.760
Hospital admissions				
All-cause hospital admissions	2,494 (0.35 [0.33-0.36])	1,204 (0.28 [0.26-0.30])	0.91 (0.84-1.00)	0.044
All CVD hospital admissions	1,167 (0.16 [0.15-0.17])	661 (0.15 [0.14-0.17])	1.07 (0.96-1.19)	0.233
Unplanned CVD hospital admissions	703 (0.10 [0.09-0.11])	342 (0.08 [0.07-0.09])	1.03 (0.89-1.19)	0.717
ED presentations				
All-cause ED presentations	1,262 (0.32 [0.29-0.34])	652 (0.29 [0.25-0.32])	0.98 (0.85-1.14)	0.816
All CVD ED presentations	470 (0.12 [0.11-0.13])	285 (0.13 [0.11-0.15])	1.10 (0.91-1.33)	0.339
Unplanned CVD ED presentations	470 (0.12 [0.11-0.13])	284 (0.13 [0.10-0.15])	1.09 (0.90-1.33)	0.360

Abbreviations as in Table 1.

a Number of events across all patients (number of events per patient within 6 months [95% CI]).

**Figure 2:**
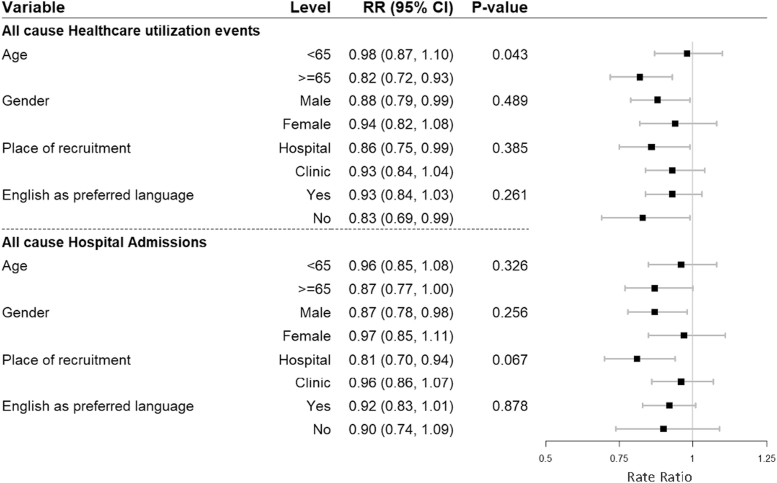
Adjusted Interaction Analysis Adjusted interaction analysis of patient variables by treatment effect on all-cause health care utilization events and hospital admissions, n = 11,542.

There was a significant interaction between age and intervention group on all-cause health care service events (*P* = 0.043), indicating the effectiveness of the intervention was greater in the older age group (Figure 2). Among patients aged 65 years and older, intervention participants experienced less events (RR: 0.82, [95% CI: 0.72-0.93]). In contrast among those aged <65 years there was no significant difference in rate of health care utilization (RR: 0.98 [95% CI: 0.87-1.10]). Place of recruitment hospital vs clinic numerically appeared to contrast, with intervention participants recruited in hospital appearing to benefit more from the text message support. There were lower rates of health care utilization events and rehospitalizations among hospital-recruited participants compared to clinic-recruited participants (Figure 2), with the interaction *P* value close to but not reaching significance for rehospitalizations (RR: 0.81 [95% CI: 0.70, 0.94] vs RR: 0.96 [95% CI: 0.86, 1.07]; *P* = 0.067).

### Impact on healthy lifestyle behaviors

**Figure 3:**
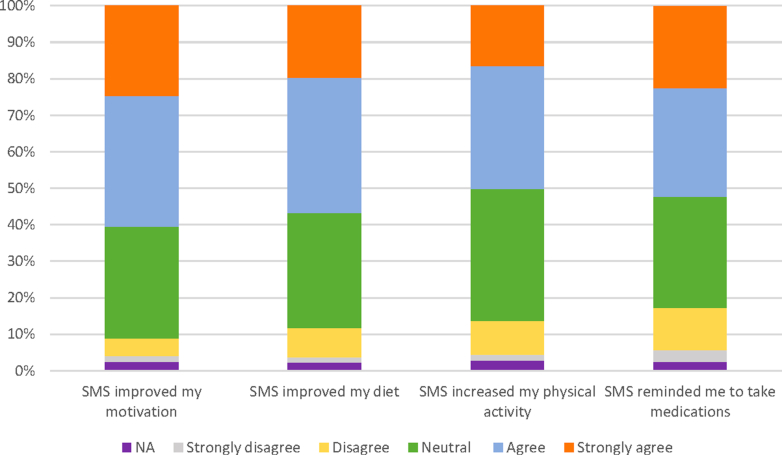
Participant Program Feedback Participant feedback on program usability and impact on behavior change, n = 1,230.

The HeartHealth program led to healthy lifestyle behavior changes for the majority of participants. Of the participants, 60.6% (746/1,230) reported that the program improved their motivation to lead a healthy lifestyle, 56.8% (699/1,230) reported that the program improved the healthiness of their diet, 50.1% (616/1,230) reported that the program increased their physical activity, and 52.3% (643/1,230) of participants reported that the program reminded them to adhere to their medication regime (Figure 3).

### Implementation measures

The majority of participants found the delivery of the HeartHealth program to be appropriate; 84.8% (1,043/1,230) found the program useful (Agreed: 45%, Strongly agreed 39.8%), 95.0% (1,168/1,230) found the SMS messages easy to understand (Agreed: 38.7%, Strongly agreed 56.3%), and 89.8% (1,104/1,230) of participants reported that the language of the SMS messages was ideal for the program. Of participants, 84.8% (1,043/1,230) and 69.3% (853/1,230) reported that the SMS frequency and program duration was ideal, respectively. Furthermore, 70.6% (868/1,230) of participants reported that the timing of the SMS delivery was appropriate (Agreed: 55.2%, Strongly agreed 15.4%).

**Figure 4:**
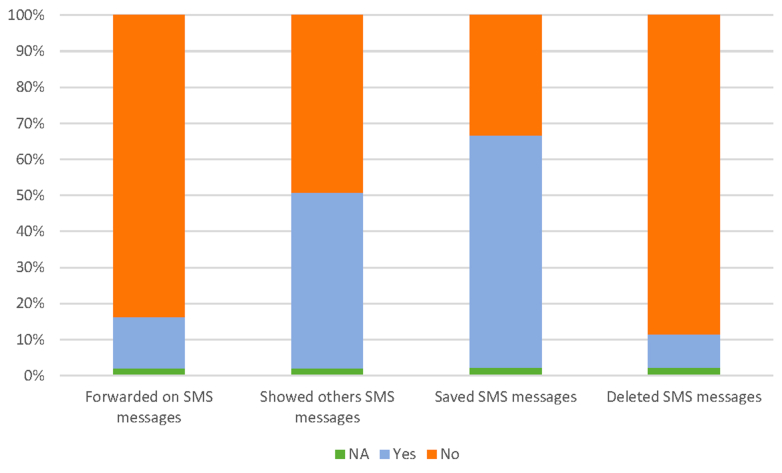
Participant Interaction With Delivered SMS Messages Participant interaction with delivered SMS messages; responses include whether participants forwarded the message, showed it to others, saved it, or deleted the SMS, n = 1,230. SMS = short message service.

There was a high level of patient engagement with the intervention among the majority of participants. 86.5% (1,064/1,230) of participants read >75% of the delivered SMS messages (90%-100%: 69.0%, 75%-90%: 17.5%) (Supplemental Figure 1). Many participants shared the messages with other people (showed others: 600/1,230; 48.8%; forwarded messages: 173/1,230; 14.1%) (Figure 4). For future use, 64.3% (791/1,230) saved the messages, whereas only 9.2% (113/1,230) of participants deleted the delivered messages (Figure 4).

7,255 follow-up enrollment phone calls were made when patients had not responded to the initial SMS program invitation. Most participants enrolled or indicated that they would enroll in the program during or after the follow-up phone calls. The main reasons for participants not enrolling postphone calls were due to not answering the phone, not being interested in the program, patients unable to speak English, or phone connectivity and user issues (Supplemental Table 1).

## Discussion

In this nonrandomized controlled comparison of the implementation of a semipersonalized text messaging program providing cardiovascular education, general health supportive information, and support for healthy lifestyle change compared with usual care, program participants had about a 10% lower rate of repeat health care service events. Participants were cardiovascular inpatients and attendants at cardiovascular outpatient clinics during the COVID-19 pandemic in Australia. Program participants were less likely to have any health care service event (composite of hospital admissions and ED presentations) in the 6 months after discharge, with people aged 65 years and older significantly more likely to benefit from the program, and data also suggesting hospital-recruited patients were more likely to benefit compared to clinic-recruited patients. Program participants did have lower levels of some comorbidities (Central Illustration); however, analyses are adjusted, and while adjusting did attenuate differences, health care utilization remained significantly lower among program participants. This study therefore suggests that opting into a customized text message-based postdischarge education, and support program was associated with less postdischarge health care utilization but does not infer causality.

**Central Illustration:**
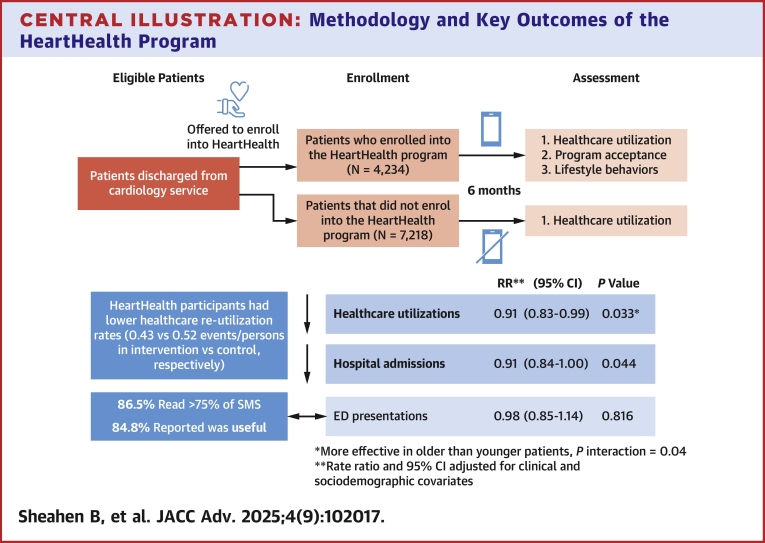
Methodology and Key Outcomes of the HeartHealth Program Outline of the HeartHealth program and assessment, as well as key outcomes on health care service utilization and participant feedback on the program. ED = emergency department.

The mechanism through which the HeartHealth program was associated with decreased incidence of health care service events is likely complex. It is possible that it was a result of improved health and cardiovascular profiles through implementing healthy behaviors and improved cardiovascular and health knowledge through the program aiding in frequent reminders to adhere to medications and healthy behaviors and improved navigation of the health care system through program participants having opportunities to ask questions via text message. The greater benefit among people aged 65 years and older may be due to differences in health care attitudes, abilities to navigate health care, and higher rates of health care utilization, usually or by chance. The HeartHealth intervention was based on the intervention used in the TextMe study.^12^ TextMe addressed the efficacy of an SMS lifestyle-focused support program in reducing cardiovascular risk factors, which demonstrated improvements in low density lipoprotein cholesterol levels, blood pressure, physical activity, and smoking rates. Additionally, other text messaging interventions have improved cardiovascular risk factor profiles, improved healthy lifestyle activities, and improved motivation to lead a healthier lifestyle.^13^^,^^14^ The HeartHealth program evaluation results aligned with this previous research, highlighting that most intervention participants reported an increased motivation to lead a healthier lifestyle, implemented a healthier diet, increased their physical activity levels, and improved medication adherence. Future research to explore objective reasons for program effectiveness is needed.

This study builds on a growing body of literature on the utility of a postdischarge text messaging program to improve secondary prevention of CVD and support patients in a community setting.^25^, ^26^, ^27^ Some randomized controlled trials have evaluated the impact of text messaging programs upon hospital readmissions in specific cardiovascular populations, such as acute coronary syndrome patients and chronic heart failure patients. These programs differed in their approach, with 1 study focusing on medication adherence^26^ and the other 2 focusing on specific support on heart failure^27^ or acute coronary syndrome^25^ management. The findings were mixed, with some finding no significant difference^25^^,^^26^ and others finding an associated reduced hospital readmission rate.^27^ Furthermore, a recent cohort study of 1,885 patients with a mean age of 62.9 years, was conducted examining the impact of a text messaging program in postdischarge patients of any medical cause, not limited to just cardiovascular patients.^28^ The intervention was conducted in a single primary care practice and compared with a control practice within Philadelphia, USA. It was found that a 30-day text messaging program posthospitalisation led to a decrease in health care utilization events (ED presentations or hospital admission), with the intervention group having 41% lower odds of a health care utilization event within 30 days. Interestingly, like our study, while health care utilization events and hospital admission rates were significantly lower between intervention and control groups, ED presentations were not. It is important for health service providers to know whether text message-based reminder and support programs after discharge are associated with increases or decreases in subsequent health care utilization. The findings of this HeartHealth evaluation lend strength to the notion of the benefit of simple text message-based support postdischarge in diverse settings. Previous modeled health economic evaluation studies indicate text message-based interventions are likely more than cost-effective and indeed cost-saving.^15^ The current study provides direct data on reduction in health care utilization.

### Clinical implications

Text message programs in cardiovascular patients have been shown to be effective in improving healthy lifestyle behaviors, improving CVD risk factor profiles, and being well accepted by users. A key feature of mobile health interventions is the potential for scalability by virtue of ease of use, affordability, high prevalence of mobile phone owners, and ability to deliver personalized services. The adoption of mobile health technologies has been on the rise over recent years in the wake of the COVID-19 pandemic, increased focus on telehealth, and the rising burden on health care services,^29^ but to date, there has been minimal scientific data on effectiveness on health care service impact. This study helps address this gap in evidence to show that in real-world clinical practice, the delivery of a text message support program is associated with an improved pattern of lower health care service utilization in cardiovascular patients. For policymakers and health systems, the potential for improving population health and decreasing health care services is evident. Text messaging programs postdischarge for cardiovascular patients are likely to improve patient outcomes and health care service burden while being scalable at low cost with low resource requirements.

### Strengths and limitations

The strength of this study is that it describes the real-world implementation and impact of the HeartHealth program; however, this study has some limitations to be considered. First, the participants were not randomized; the intervention was composed of those who enrolled in the HeartHealth program, whereas the control group was those who did not enroll. This in-time comparator group was selected to avoid a pre-post comparison with the period prior to the COVID-19 pandemic, as studies have shown the impact of COVID-19 on hospitalization and emergency department presentation rates.^21^^,^^22^ As a result of this methodology, there were discrepancies in comorbidity and demographic variables between the 2 cohorts. To account for discrepancies between the groups, all analyses were adjusted for comorbidity and demographic variables; however, intrinsic factors such as patient motivation to improve health were not able to be quantified and adjusted for. Second, health care service events were only collected from WSLHD hospitals. Although it is probable that most health care service events involving these participants took place within WSLHD, we lacked access to data from other local health districts in New South Wales to verify this. Third, this study was carried out within a single local health district, raising questions about the generalizability of observed benefits to a broader population. Nonetheless, it is noteworthy that the WSLHD community embodies diverse economic, social, and cultural backgrounds,^24^ underscoring the potential relevance of these findings to a wider context. Fourth, the data source utilized for the collection of patient information did not contain details on key social determinants of health such as income, employment status, education levels, and social support and was unable to stratify medical conditions by severity, thereby preventing further adjusted analysis. However, key sociodemographic factors that would likely influence SMS message engagement, such as preferred language, country of birth, gender, and age, were collected and adjusted for in all analyses. Fifth, due to the retrospective nature of the study design, data on lifestyle change was limited preventing further rigorous evaluation of behavior change.

## Conclusions

Our study demonstrated that the use of a postdischarge text messaging program in cardiovascular patients was associated with a decreased need for health care service utilization. The scalability of the HeartHealth program enables its delivery to larger populations at risk, presenting significant potential for benefiting the overall population. Future studies with a randomized control design should be conducted to validate the effectiveness of the HeartHealth program in a real-world clinical setting. There is also a need for a formal cost-effectiveness evaluation to determine if the program represents a good value.

Perspectives **COMPETENCY IN PATIENT CARE AND SYSTEMS-BASED PRACTICE:** The real-world clinical program, HeartHealth, was associated with decreased health care service use in cardiovascular patients and improved lifestyle behaviors while being acceptable and engaging. Program assessment using a randomized control design in a real-world clinical setting is required to validate these findings.

**TRANSLATIONAL OUTLOOK:** The HeartHealth program is likely to improve both patient outcomes and health care service burden, while being scalable with low cost and resource requirements. For policymakers and health systems, the potential clinical benefits are evident; however, a formal cost-effectiveness evaluation should be conducted to determine if the program represents a good value.

## Funding support and author disclosures

The HeartHealth program was funded by the 10.13039/100014467WSLHD. This was supplemented, and analysis was supported by an 10.13039/501100000925NHMRC investigator grant (grant number: APP1195326). The authors have reported that they have no relationships relevant to the contents of this paper to disclose.
