# Supplementary material for: Implementation and Impact of Postdischarge Support of Cardiovascular Patients Using Text Messages The HeartHealth Program
Source: JACC Adv. 2025 Aug 6;4(9):102017. doi: 10.1016/j.jacadv.2025.102017 (PMC12351332; doi:10.1016/j.jacadv.2025.102017)
Supplement: Supplementary data [file mmc1.docx]

**Supplemental Figure 1**: Participant engagement with reading SMS messages

**Supplemental Table 1**: Patient responses to follow-up enrolment phone call

| **Responses** | **n (%)** |
| --- | --- |
| Patient did not answer the phone | 1835 (25.3%) |
| Enrolled during phone call | 1386 (19.1%) |
| Enrolled prior to phone call | 1356 (18.7%) |
| Interested in the program and the patient will self-enrol | 1109 (15.3%) |
| Patient not interested | 708 (9.7%) |
| Patient could not speak English | 333 (4.6%) |
| The enrolment link was resent to the patient | 164 (2.3%) |
| Patient’s phone was not connected | 117 (1.6%) |
| Other | 107 (1.5%) |
| Patient was not text savvy | 91 (1.2%) |
| Wrong number provided | 49 (0.7%) |
